# Heterogeneity among tumors with acquired resistance to EGFR tyrosine kinase inhibitors harboring *EGFR*‐T790M mutation in non‐small cell lung cancer cells

**DOI:** 10.1002/cam4.4504

**Published:** 2022-01-14

**Authors:** Yuki Katayama, Tadaaki Yamada, Shinsaku Tokuda, Naoko Okura, Naoya Nishioka, Kenji Morimoto, Keiko Tanimura, Yoshie Morimoto, Masahiro Iwasaku, Mano Horinaka, Toshiyuki Sakai, Kenji Kita, Seiji Yano, Koichi Takayama

**Affiliations:** ^1^ Department of Pulmonary Medicine Graduate School of Medical Science Kyoto Prefectural University of Medicine Kyoto Japan; ^2^ Department of Molecular‐Targeting Cancer Prevention Graduate School of Medical Science Kyoto Prefectural University of Medicine Kyoto Japan; ^3^ Division of Medical Oncology Cancer Research Institute Kanazawa University Kanazawa Japan

**Keywords:** dacomitinib, EGFR‐T790M mutation, gefitinib, tumor heterogeneity

## Abstract

*EGFR*‐T790M mutation is a major mechanism underlying acquired resistance to first‐ and second‐generation EGFR tyrosine kinase inhibitors (EGFR‐TKIs) in lung cancer with mutated *EGFR*. However, differences in the biological characteristics of T790M tumors based on treatment regimens with each generation of EGFR‐TKI are not fully understood. We established cell lines with acquired resistance harboring *EGFR*‐T790M mutation derived from xenograft tumors treated with each generation of EGFR‐TKI and examined their biological characteristics with respect to third‐generation EGFR‐TKI osimertinib sensitivity. Second‐generation EGFR‐TKI dacomitinib‐resistant cells with T790M‐exhibited higher sensitivity to osimertinib than first‐generation EGFR‐TKI gefitinib‐resistant cells with T790M via inhibition of AKT and ERK signaling and promotion of apoptosis. Furthermore, gefitinib‐resistant cells showed enhanced intratumor heterogeneity accompanied by genomic instability and activation of alternative resistance mechanisms compared with dacomitinib‐resistant cells; this suggests that the maintenance of EGFR dependency after acquiring resistance might depend on the type of EGFR‐TKI. Our results demonstrate that the progression of tumor heterogeneity via both genetic and non‐genetic mechanisms might affect osimertinib sensitivity in tumors with acquired resistance harboring *EGFR*‐T790M mutation.

## Graphical abstract

EGFR‐TKI‐resistant EGFR T790M‐mutant tumors were developed in vivo. A difference in sensitivity to osimertinib was observed between the T790M tumors. A difference in genetic and epigenetic intratumor heterogeneity was observed.

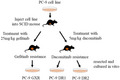

## 1 BACKGROUND

Lung cancer is the leading cause of cancer deaths worldwide.^1^ Recently, the development of molecular‐targeted therapy has markedly improved clinical outcomes in patients with non‐small cell lung cancer (NSCLC) who show expression of driver oncogenes. For example, patients with epidermal growth factor receptor (*EGFR*) mutations, including exon 19 deletion and L858R point mutation in exon 21, showed improved outcomes with EGFR tyrosine kinase inhibitors (EGFR‐TKIs).^2^, ^3^ To date, three generations of EGFR‐TKIs have been developed.^4^, ^5^, ^6^ However, most patients acquire resistance to initial EGFR‐TKIs after various intervals. The resistance mechanisms include “on‐target” effects, such as EGFR resistance mutations, and “off‐target” effects, such as activation of bypass signals (amplification of *MET* and *HER2*, and mutations in *BRAF* and *PIK3CA*), epithelial–mesenchymal transition (EMT), and transformation to small cell lung cancer.^7^, ^8^ Of these, the *EGFR*‐T790M mutation, an “on‐target” resistance mechanism, is the most common mechanism underlying acquired resistance to first‐ and second‐generation EGFR‐TKIs.^7^, ^9^ The third‐generation EGFR‐TKI osimertinib shows better progression‐free survival than platinum‐based chemotherapy against EGFR resistance mutations, such as *EGFR*‐T790M.^10^ Osimertinib has shown better outcomes than the first‐generation EGFR‐TKIs as a first‐line treatment for patients with advanced *EGFR*‐mutated NSCLC. Hence, osimertinib has been approved for managing untreated and *EGFR*‐T790M‐mutated advanced NSCLC in the United States and other countries.^11^ Nonetheless, molecular‐targeted therapy for acquired osimertinib resistance has not yet been developed. Treatment with the second‐generation EGFR‐TKI afatinib followed by osimertinib showed favorable outcomes in terms of time‐to‐treatment failure and overall survival in the presence of T790M mutation.^12^ Therefore, sequential treatment with initial EGFR‐TKIs followed by osimertinib is a promising first‐line treatment strategy owing to an increase in the interval showing durable response to EGFR‐TKIs and relatively low toxicity compared to cytotoxic chemotherapy.

Intratumor heterogeneity is related to the promotion of tumor recurrence and intrinsic drug resistance. In patients with NSCLC harboring *EGFR* mutations, heterogeneous pretreatment tumor evolution is closely associated with the rapid development of resistance to EGFR‐TKIs.^13^ NSCLC patients with concurrent *EGFR*‐T790M mutation and *MET* amplification showed poorer outcomes to osimertinib treatment than those with *EGFR*‐T790M mutation alone.^14^ Therefore, the evolution of intratumor heterogeneity is predicted to play crucial roles in sensitivity to osimertinib in resistant tumors with *EGFR*‐T790M mutation. Although both first‐ and second‐generation EGFR‐TKIs result in the development of *EGFR*‐T790M‐resistant clones, the range of signaling blockade differs. First‐generation EGFR‐TKIs, including gefitinib, inhibit EGFR, and second‐generation EGFR‐TKIs, including dacomitinib, inhibit pan‐HER family proteins.^4^, ^5^, ^6^ Therefore, each generation of EGFR‐TKIs might elicit a different resistant tumor phenotype.

To verify these hypotheses, we established *EGFR*‐T790M‐positive cells derived from preclinical xenograft tumors treated with different EGFR‐TKIs (gefitinib or dacomitinib). We evaluated the intratumor heterogeneity of these clones regarding osimertinib sensitivity and the molecular and biological aspects in NSCLC cells harboring *EGFR* mutations.

## 2 METHODS

### 2.1 Cell cultures and reagents

The PC‐9 cell line was obtained from the Riken Cell Bank (Ibaraki, Japan). The PC‐9 GXR cells harboring deletions in *EGFR* exon 19 and the T790M mutation were developed at Kanazawa University (Ishikawa, Japan) from PC‐9 cell xenograft tumors in severe combined immunodeficiency (SCID) mice that had acquired resistance to gefitinib. PC‐9 DR1 and PC‐9 DR2 cells, with deletions in *EGFR* exon 19 and the T790M mutation, were developed at Kyoto Prefectural University of Medicine (Kyoto, Japan) from PC‐9 cell xenograft tumors in SCID mice with acquired resistance to dacomitinib. All cell lines were maintained in Roswell Park Memorial Institute (RPMI) 1640 medium (Gibco) supplemented with 10% fetal bovine serum, penicillin (100 U/ml), and streptomycin (50 g/ml) in a humidified CO_2_ incubator at 37°C. All cells were passaged for less than 3 months before being replaced with frozen, early‐passage stocks. The cells were regularly screened for mycoplasma using a MycoAlert Mycoplasma Detection Kit (Lonza). The cell lines were authenticated via DNA fingerprinting. Gefitinib, osimertinib, and dacomitinib were obtained from Selleckchem.

### 2.2 Cell line‐derived xenograft models

Suspensions of 5 × 10^6^ cells were injected subcutaneously into the flanks of 5‐week‐old male C.B‐17/Icr‐scid/scidJcl mice with SCID (Clea Japan), as previously described.^15^ Once the mean tumor volume reached approximately 100–200 mm^3^, each mouse was injected with the indicated treatment in the PC‐9 cell line‐derived xenografts. Each treatment group consisted of six mice. Drugs (25 mg/kg gefitinib or 5 mg/kg dacomitinib) were administered 7 days a week via oral gavage, as described in previous reports.^16^, ^17^ Body weight was measured twice weekly, and the general condition was monitored daily. Tumor dimensions were measured twice weekly using calipers, and their volumes were calculated using the following formula: volume = width^2^ × length/2. This study was approved by the institutional review board at University Hospital, Kyoto Prefectural University of Medicine (approval no.: M29‐529). According to the institutional guidelines, surgery was performed after anesthetizing the animals with sodium pentobarbital and efforts were made to minimize animal suffering.

### 2.3 Cell viability assay

Tumor cells (2–3 × 10^3^ cells/100 µl/well) in RPMI‐1640 medium supplemented with 10% fetal bovine serum were seeded in 96‐well plates and cultured with the indicated compound for 72 h. Cell viability was determined using the MTT dye reduction method, as previously described.^15^ The percentage of growth was determined relative to that in untreated controls. The seeded cells were stained with crystal violet and examined visually. Experiments were repeated at least thrice.

### 2.4 Western blotting

Twenty‐five microgram of protein samples were resolved using SDS–polyacrylamide gel electrophoresis (Bio‐Rad Laboratories). The western blotting was performed as previously described.^15^ Each experiment was performed independently at least three times. Antibodies used in this study are presented in Table S1.

### 2.5 *EGFR* allele quantification

Droplet digital PCR was performed using an LBx probe for the *EGFR* exon 19 del or T790M mutation. Samples were quantified using a QX200 droplet reader (Bio‐Rad Laboratories). The distribution of the wildtype, exon 19 del, and/or T790M alleles was determined by Riken Genesis. There was individual experiment.

### 2.6 siRNA transfection

Cells were transfected with Duplexed Silencer Select siRNAs for *EGFR* (s564 and s565; Invitrogen, Carlsbad, CA, USA) using Lipofectamine RNAi‐MAX (Invitrogen) according to the manufacturer's instructions. The Silencer^®^ Select siRNA for negative control #1 (Invitrogen) was used as the scrambled control. *EGFR* knockdowns were confirmed using western blotting. Each sample was analyzed independently at least thrice.

### 2.7 Apoptosis analysis

Cells were harvested 48 h after treatment with 100 nM osimertinib, washed twice with ice‐cold phosphate‐buffered saline (PBS), and incubated with Annexin V‐fluorescein isothiocyanate and propidium iodide (PI) for 15 min at room temperature. The cells were analyzed using BD Accuri™ C6 Plus Flow Cytometer and the FlowJo^®^ software. For each culture, at least 1 × 10^4^ events were recorded.

### 2.8 Cell cycle analysis

Cells were harvested 48 h after treatment with 100 nM osimertinib, washed twice with ice‐cold PBS, collected by centrifugation, and resuspended at 1 × 10^6^ cells/ml in PI staining buffer (0.1% Triton X‐100 and 50 µg/ml PI in PBS). The cells were analyzed using BD Accuri™ C6 Plus Flow Cytometer and the FlowJo^®^ software. For each culture, at least 1 × 10^4^ events were recorded.

### 2.9 Soft agar assays

Colony formation of PC‐9 and resistant cells was assessed using the CytoSelect 96‐Well In Vitro Tumor Sensitivity Assay (Soft Agar Colony Formation) kit (Cell BioLabs). Briefly, 50 µl of base agar matrix was dispensed into each well of a 96‐well tissue culture plate. A total of 5,000 cells in 75 µl of cell suspension agar matrix were dispensed into each well, and 50 µl of culture medium was added to each well containing various drugs, as indicated. Fresh medium with drugs was added every 72 h. After 10 days of incubation, the matrix was solubilized, and MTT reagent was added to each well. The absorbance was measured using a SpectraMax fluorometer at 570 nM.

### 2.10 Whole‐exome sequencing

All reads were quality‐checked using FastQC (ver0.11.8). Using Trim Galore (ver0.6.4), we discarded short reads and reads with insufficient base qualities. The trimming process was conducted with the default settings. The trimmed reads were aligned to the reference genome (GRCh38) using BWA‒MEM (ver0.7.17). The index files required by BWA were generated separately. Mapped reads were sorted using SAMtools. Using Picard MarkDuplicates (ver2.22.9), reads sequenced together were marked, followed by marking duplicate reads (possibly PCR duplicates). Base recalibration was conducted in the final step of postprocessing using BaseRecalibrator and ApplyBQSR (GATK ver4.1.9.0). Somatic point mutations and indels were called simultaneously, and the results were stored as a VCF file by running Mutect2 Tumor only mode (GATK ver4.1.9.0). Tumor mutation burden (TMB) was defined as the total number of “non‐silent mutations” per million base pairs of tumor DNA in each sample. The gene length was calculated from exon length defined by SureSelect v6 (Agilent Technology). Chromosome instability score (CIS) was defined as “the altered fraction of the genome” and calculated using cnvkit (ver0.9.8) and pureCN (ver1.20.0). Mutant‐allele tumor heterogeneity (MATH) was calculated as an index of intratumor heterogeneity. MATH score was calculated as 100 times the median absolute deviation/median of the variant allele frequencies, which were calculated as the ratio of alternate allele observations to the read depth at each position. There was individual experiment.

### 2.11 Microarray analysis

The GeneChip^®^ WT PLUS Reagent Kit was used to synthesize cDNA with biotin‐labeled 3′‐end from 100 ng of total RNA according to the protocol recommended by Thermo Fisher Scientific. Hybridization of biotin‐labeled cDNA and Clariom S Array (Thermo Fisher Scientific) was performed. After washing and staining, signals were detected using a scanner. Gene set enrichment analysis was used to investigate the enriched functions of specifically expressed genes. The specifically expressed genes were ranked according to fold changes compared with hallmark gene sets, which summarized and represented specific, well‐defined biological states or processes and displayed coherent expression. The number of random sample permutations was set at 1000. The significance threshold was set at *p* < 0.05, with a false discovery rate <0.25.

### 2.12 Immunofluorescence microscopy

Cells were cultured on 12 mm‐diameter Transwell filter inserts with a 0.4‐μm pore size (Corning) for 6 days, fixed in 3.7% formaldehyde at room temperature for 20 min, permeabilized in a solution of 0.2% (w/v) Triton X‐100 (Nacalai) for 60 min, and incubated with 2% bovine serum albumin in PBS for 60 min. The cells were then incubated with a primary antibody for 60 min (see Table S1 for antibodies and dilutions used), followed by incubation with a fluorescently labeled secondary antibody. Samples were imaged using a Zeiss LSM900 confocal microscope.

### 2.13 Migration assay

For Transwell migration assays, approximately 5.0 × 10^4^ cells in 100 μl of serum‐free RPMI‐1640 were seeded onto filter inserts. Medium containing 10% FBS was placed in the lower chamber as a chemoattractant to encourage cell migration. After 24 h of incubation, inserts were fixed and stained with Giemsa stain. The non‐migrated cells were removed using cotton swabs and air‐dried. Membranes were photographed using a microscope at ×100 magnification. We selected three random views per membrane to count the number of cells.

### 2.14 Statistical analysis

Data from the MTT assays are expressed as mean ± SD. The differences were statistically analyzed with one‐way ANOVA and two‐way ANOVA using Prism 8.0 (GraphPad Software). Two‐sided *p*‐values <0.05 were considered statistically significant.

## 3 RESULTS

### 3.1 Characteristics of PC‐9‐derived cell lines with acquired *EGFR*‐T790M mutation from in vivo xenograft models

**FIGURE 1:**
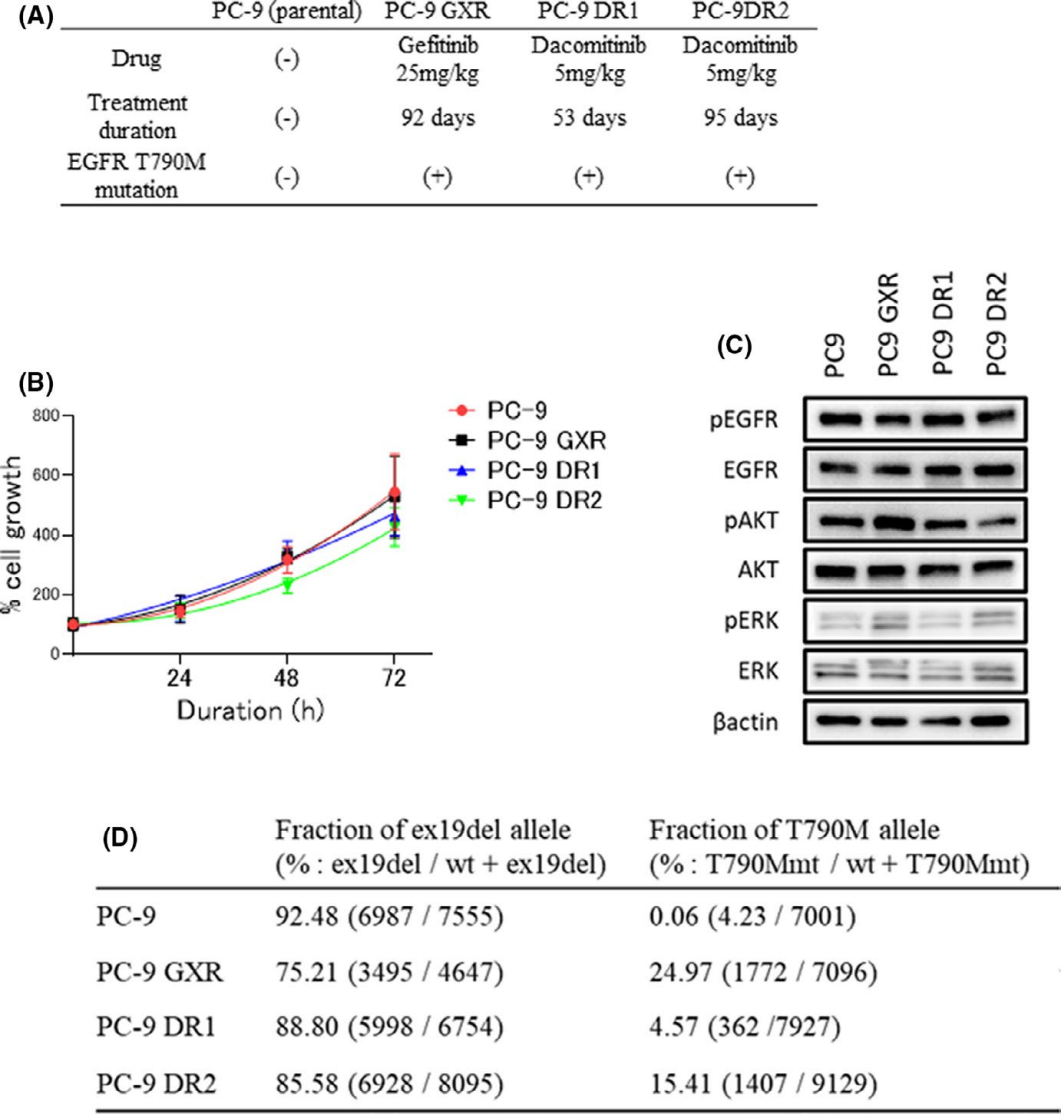
Characteristics of PC‐9‐derived cell lines with acquired *EGFR*‐T790M resistance mutation from in vivo xenograft models. (A) Treatment regimen for the development of drug‐resistant cell lines: gefitinib‐resistant PC‐9 GXR and dacomitinib‐resistant PC‐9 DR1 and PC‐9 DR2 derived from PC‐9 cells. (B) Cells were incubated for the indicated times, and their viability was assessed using MTT assay. (C) Protein expression in PC‐9 non‐resistant and resistant cells was analyzed using western blotting with the indicated antibodies. The housekeeping gene was *ACTB*. (D) Proportion of *EGFR* exon 19 del and T790M alleles analyzed using digital PCR

To evaluate *EGFR*‐T790M clones with resistance to different EGFR‐TKIs, we established gefitinib‐ or dacomitinib‐resistant tumors with *EGFR*‐T790M mutation from an in vivo xenograft model. After resistance acquisition, tumors were resected, and gefitinib‐ and dacomitinib‐resistant cells were cultured in vitro (Figure 1A). We confirmed the development of *EGFR*‐T790M mutation in one gefitinib‐resistant clone, PC‐9 GXR, and two dacomitinib‐resistant clones, PC‐9 DR1 and PC‐9 DR2 (Figure S1). The growth curves of PC‐9 GXR and PC‐9 DR1 cells were similar to that of PC‐9 cells, whereas PC‐9 DR2 cells demonstrated a slightly slower growth rate (Figure 1B). Western blot analysis showed similar expression of EGFR and related proteins in PC‐9, PC‐9 GXR, PC‐9 DR1, and PC‐9 DR2 cells (Figure 1C). The number of genetic alterations in *EGFR* was analyzed using droplet digital PCR. The allele frequency of *EGFR* exon 19 deletion in PC‐9 GXR cells was lower than that in PC‐9 cells, while in PC‐9 DR1 and PC‐9 DR2 cells, it did not differ. The allele frequency of the *EGFR* exon 20 T790M mutation was higher in PC‐9 GXR cells than in PC‐9 DR1 and PC‐9 DR2 cells (Figure 1D). Hence, treatment with different types of EGFR‐TKIs might contribute to the diversity in the allele frequency of *EGFR* in resistant tumors with *EGFR*‐T790M mutation.

### 3.2 Diversity of drug sensitivity in PC‐9‐derived cell lines with *EGFR*‐T790M mutation

**FIGURE 2:**
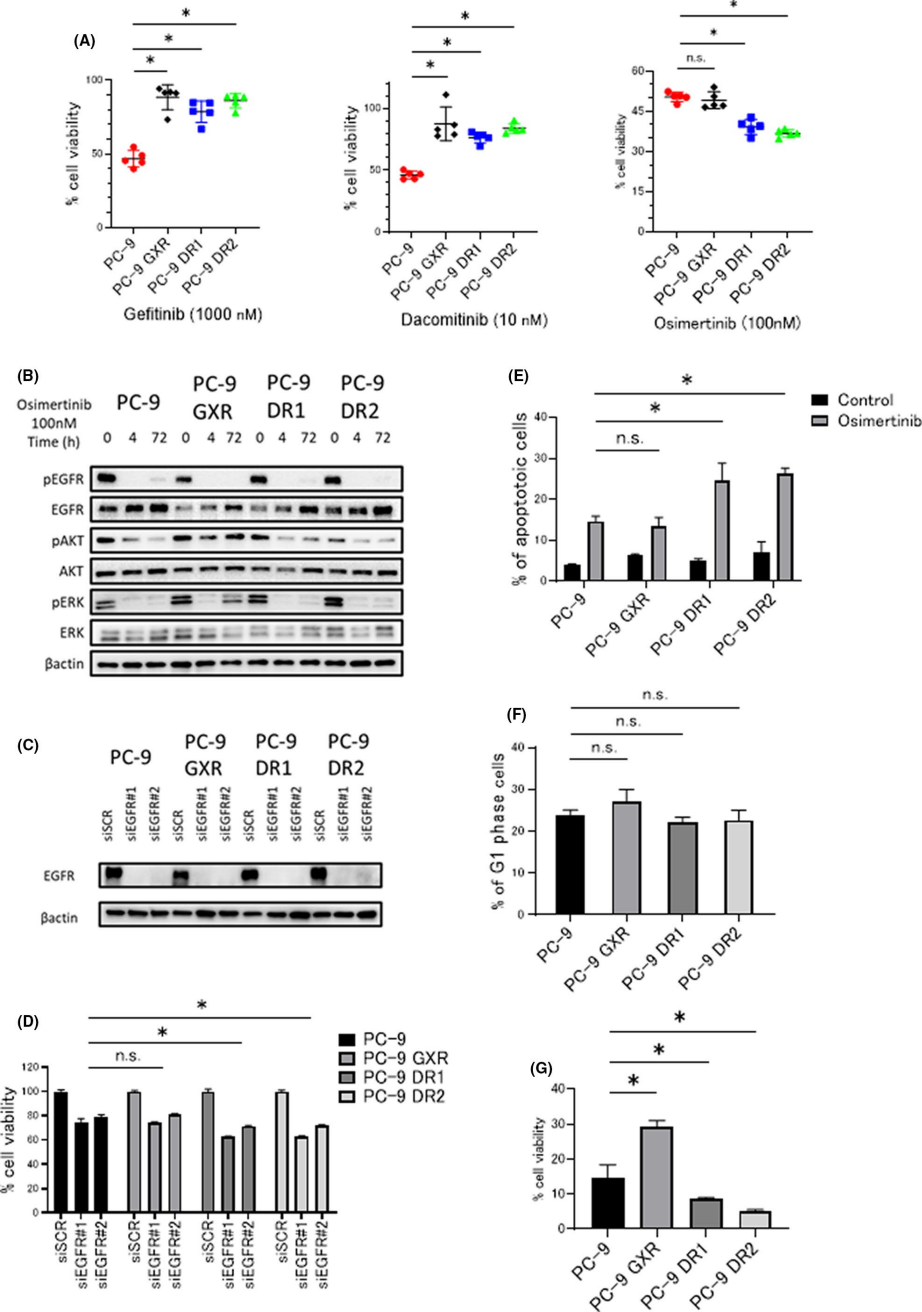
Evaluation of osimertinib sensitivity and molecular and biological characterization of *EGFR*‐T790M‐mutated resistant cells. (A) Growth inhibition of PC‐9 and resistant cells treated with 1000 nM gefitinib, 10 nM dacomitinib, or 100 nM osimertinib for 72 h assessed using MTT assay. *p < 0.001 compared with PC‐9 cells, one‐way ANOVA. (B) Cells were incubated with osimertinib (100 nmol/L) for 4 or 72 h and lysed. The indicated proteins were detected using western blotting. (C) Cells treated with nonspecific control siRNA or *EGFR*‐specific siRNAs (#1 and #2) were incubated for 72 h and lysed. The indicated proteins were detected using western blotting. (D) Cells treated with nonspecific control siRNA or *EGFR*‐specific siRNAs (#1 and #2) were incubated for 72 h, and cell viability was detected using MTT assay. *p < 0.001 compared with PC‐9 cells, two‐way ANOVA. (E) Proportion of apoptotic cells in PC‐9 and resistant cell lines after treatment with 100 nM osimertinib for 48 h was determined using Annexin V‐ fluorescein isothiocyanate and PI staining. *p < 0.01 compared with PC‐9 cells treated with osimertinib, two‐way ANOVA. (F) Number of cells in the G1 phase in PC‐9 and resistant cell lines increased after treatment with 100 nM osimertinib for 48 h. n.s., not significant, one‐way ANOVA. (G) Growth inhibition of long‐term culture model with soft agar assay of PC‐9 and resistant cells treated with 100 nM osimertinib for 10 days. *p < 0.01 compared with PC‐9 cells treated with osimertinib, one‐way ANOVA

We next examined the sensitivity of PC‐9 and resistant cells with *EGFR*‐T790M mutation derived from PC‐9 cells to each generation of EGFR‐TKIs, namely, gefitinib, dacomitinib, and osimertinib. As expected, only PC‐9 cells were sensitive to gefitinib and dacomitinib. Osimertinib treatment significantly inhibited the growth of PC‐9 DR1 and PC‐9 DR2 cells compared with that of PC‐9 and PC‐9 GXR cells (Figure 2A; Figure S2). AKT phosphorylation was significantly inhibited in PC‐9 DR1 and DR2 cells treated with 100 nM osimertinib for 4 and 72 h compared with that in PC‐9 GXR cells. ERK phosphorylation was significantly inhibited in PC‐9 DR1 and DR2 cells treated with 100 nM of osimertinib for 72 h compared with that in PC‐9 GXR cells (Figure 2B; Figure S3A,B). To observe the dependency of cell viability on EGFR signaling, we examined the effect of *EGFR* knockdown using specific siRNAs. *EGFR* knockdown strongly inhibited the viability of PC‐9 DR1 and DR2 cells compared with that of PC‐9 and PC‐9 GXR cells, suggesting that the dependency on EGFR signaling might be maintained in dacomitinib‐resistant cells compared with that in gefitinib‐resistant cells (Figure 2C,D). Treatment with osimertinib for 48 h significantly increased the percentage of apoptotic cells in PC‐9 DR1 and PC‐9 DR2 cell lines compared with that in PC‐9 and PC‐9 GXR cell lines (Figure 2E; Figure S4). In contrast, the frequency of cell cycle arrest in the G1 phase was not affected by osimertinib treatment (Figure 2F; Figure S5). In colony formation assays, osimertinib treatment for 10 days significantly inhibited the growth of PC‐9 DR1 and PC‐9 DR2 cells compared with that of PC‐9 and PC‐9 GXR cells (Figure 2G).

Collectively, dacomitinib‐resistant cells with *EGFR*‐T790M mutation showed higher sensitivity to osimertinib than gefitinib‐resistant *EGFR*‐T790M‐mutated cells via inhibition of AKT and ERK signaling and promotion of apoptosis. This indicates a high dependency of dacomitinib‐resistant cells on EGFR signaling.

### 3.3 Genetic heterogeneity with *EGFR*‐T790m mutation observed in cells resistant to different EGFR‐TKIs

**FIGURE 3:**
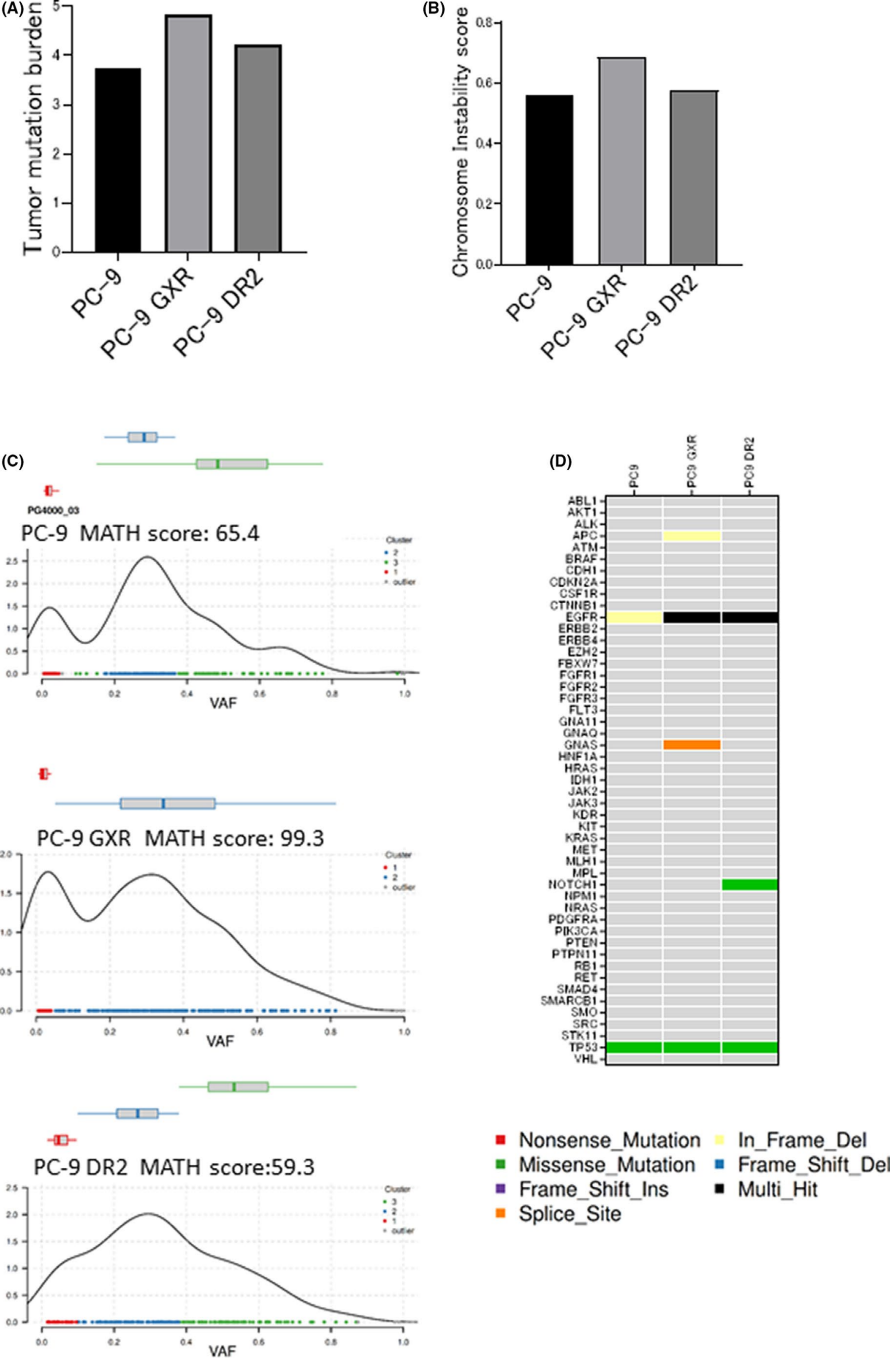
Genetic heterogeneity in PC‐9 and resistant cells with *EGFR*‐T790M mutation. (A) Tumor mutation burden of PC‐9, PC‐9 GXR, and PC‐9 DR2 cells, defined as non‐silent mutation per 1 million base pairs. (B) Chromosome instability scores of PC‐9, PC‐9 GXR, and PC‐9 DR2 cells, defined as the altered fraction of the genome. (C) MATH scores of PC‐9, PC‐9 GXR, and PC‐9 DR2 cells, defined as the ratio of the width of the distribution to the median distribution of the variant allele frequency. (D) Heatmap of major oncogene mutations in PC‐9, PC‐9 GXR, and PC‐9 DR2 cells

To evaluate tumor heterogeneity in terms of genetic mechanisms, genetic alterations between PC‐9 cells and resistant cells, PC‐9 GXR and PC‐9 DR2 cells, were examined based on TMB, CIS, and MATH scores using whole‐exome sequencing. PC‐9 GXR cells exhibited higher TMB, CIS, and MATH scores than PC‐9 and PC‐9 DR2 cells (Figure 3A–C). We next analyzed mutations in 49 major cancer‐associated genes in each cell line. In addition to *EGFR* exon 19 deletion and *TP53*, several new mutations in *APC* and *GNAS* in PC‐9 GXR cells and *NOTCH1* in PC‐9 DR2 cells were detected (Figure 3D).

Collectively, the TMB, CIS, and MATH scores were relatively high for PC‐9 GXR cells, indicating that gefitinib‐resistant cells showed higher genetic heterogeneity than dacomitinib‐resistant cells.

### 3.4 Variations in transcription activity among *EGFR*‐T790M mutant cells with acquired resistance

**FIGURE 4:**
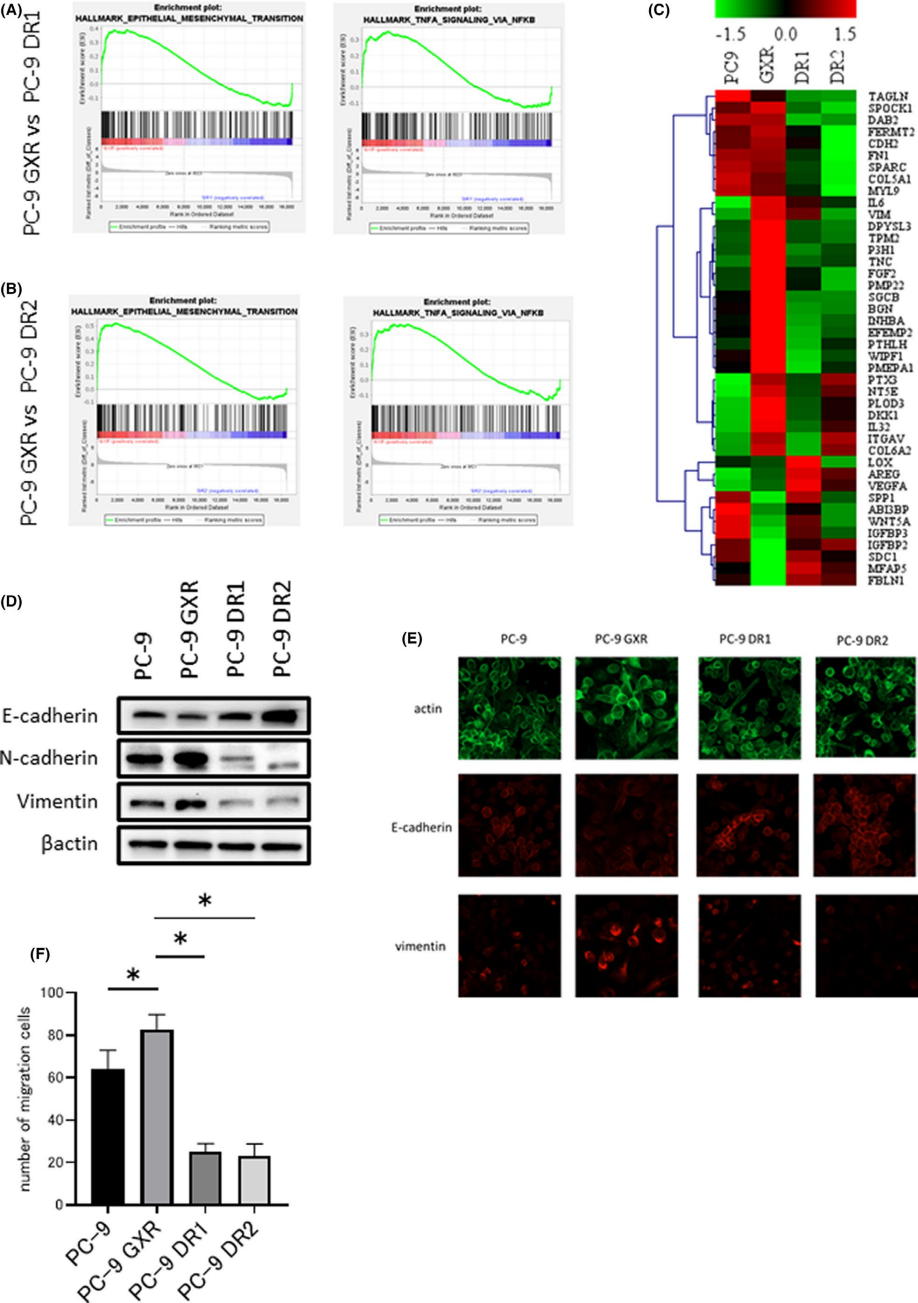
Variations in transcription activity among *EGFR*‐T790M‐mutated cells with acquired resistance. (A, B) Gene set enrichment analysis (GSEA) plot showing enrichment of common gene signatures associated with EMT and TNF‐α between PC‐9 GXR versus PC‐9 DR1 cells (A) and PC‐9 GXR versus PC‐9 DR2 cells (B). (C) Heat map of gene expression *z*‐score of the up‐ and downregulated EMT‐related genes between PC‐9 GXR and PC‐9 DR1 cells, analyzed based on the microarray data of PC‐9, PC‐9 GXR, PC‐9 DR1, and PC‐9 DR2 cells. (D) Protein expression in PC‐9 and resistant cells was analyzed using western blotting with the indicated antibodies. (E) Immunofluorescence staining of E‐cadherin, vimentin, and actin in PC‐9 and resistant cells. (F) The migration ability of PC‐9 and resistant cells was evaluated by migration assay. *p < 0.05 compared with PC‐9 GXR cells, one‐way ANOVA

To investigate the differences in transcriptomes between gefitinib‐ and dacomitinib‐resistant cells, mRNA microarray analysis was conducted. Gene set enrichment analysis indicated an increase in expression with a normalized enrichment score of 1.5‐fold or higher for PC‐9 GXR cells based on 50 hallmark gene sets consisting of genes related to biological functions (Figure S6A,B). The upregulation of EMT and TNFα signaling in PC‐9 GXR cells was remarkably enhanced compared with that in PC‐9 DR1 or DR2 cells (Figure 4A,B). A heat map of EMT‐related genes showed a different pattern for PC‐9 GXR cells than for PC‐9, PC‐9 DR1, and DR2 cells (Figure 4C). PC‐9 DR1 and PC‐9 DR2 cells showed higher expression of the epithelial marker, E‐cadherin, and lower expression of the mesenchymal markers, N‐cadherin, and vimentin, than PC‐9 and PC‐9 GXR cells (Figure 4D). Immunostaining assays demonstrated decreased E‐cadherin expression and enhanced vimentin expression in PC‐9 GXR cells and increased E‐cadherin expression in PC‐9 DR1 and PC‐9 DR2 cells compared with that in PC‐9 cells, which is consistent with western blotting results (Figure 4E). Hence, gefitinib‐resistant cells showed EMT phenotype compared with PC‐9 cells, whereas the dacomitinib‐resistant cells displayed increased E‐cadherin and decreased N‐cadherin and vimentin expression compared with PC‐9 cells. To evaluate the potency of cell migration, we next performed a migration assay. PC‐9 GXR cells displayed an increase in cell migration compared with PC‐9 DR1 and DR2 cells (Figure 4F; Figure S7).

Thus, indicators of transcriptomic activity, such as EMT and TNFα signaling, were enhanced in gefitinib‐resistant cells compared with that in dacomitinib‐resistant cells, thereby, enhancing intratumor heterogeneity.

## 4 DISCUSSION

Multiple promising strategies with various EGFR‐TKIs have been successfully developed as first‐line treatments for patients with advanced *EGFR*‐mutated NSCLC. Of these, EGFR‐TKI monotherapy and combination with anti‐angiogenetic agents have led to the emergence of *EGFR*‐T790M mutation in tumors with acquired resistance.^18^, ^19^ Questions have been raised about whether the best strategy should involve osimertinib treatment as the first‐line treatment or in a sequential regimen following the development of *EGFR*‐T790M mutation. Tumors with *EGFR*‐T790M mutation can be treated with osimertinib after they acquire resistance to first‐ and second‐generation EGFR‐TKIs. Therefore, a better understanding of T790M‐mutated tumors is essential for optimizing the clinical outcomes in patients with NSCLC harboring *EGFR* mutations.

In this study, gefitinib‐resistant *EGFR*‐T790M‐positive cells were less sensitive to osimertinib than dacomitinib‐resistant *EGFR*‐T790M‐positive cells. In the subset analysis for the LUX‐LUNG7 clinical trial, patients with NSCLC with *EGFR* mutations treated with the second‐generation EGFR‐TKI afatinib followed by osimertinib showed better outcomes than those treated with gefitinib followed by osimertinib, consistent with our preclinical observations.^12^, ^20^ The second‐generation EGFR‐TKIs, afatinib, and dacomitinib, with a broader inhibition spectrum against HER family proteins, showed better inhibitory activity against various EGFR‐activating mutations, including several compound and minor mutations, than first‐generation EGFR‐TKIs.^4^, ^5^ In this study, osimertinib treatment reduced the promotion of apoptosis and inhibition of AKT and ERK signaling in gefitinib‐resistant cells to a greater degree than in dacomitinib‐resistant cells. HER family proteins, ErbB2 and ErbB3, activate AKT and ERK signals upon acquiring resistance to specific EGFR‐targeted therapy.^21^, ^22^ Such inhibitory profiles might suppress the expansion of various resistant clones by bypassing signal activation after acquiring resistance to initial EGFR‐TKIs.^23^

A positive association between the allelic frequency of T790M mutation in a plasma sample and osimertinib response has been observed.^24^, ^25^ In the present study, the levels of the allelic frequency of T790M mutation did not correspond to the sensitivity of osimertinib between gefitinib‐ and dacomitinib‐resistant cells. The allelic frequency of *EGFR* mutations was lower in gefitinib‐resistant cells than in parental and dacomitinib‐resistant cells. Compared with the viability of gefitinib‐resistant cells, the viability of dacomitinib‐resistant cells were more highly dependent on *EGFR*, as observed upon *EGFR* knockdown. The allele frequency of *EGFR* mutations is related to the sensitivity of EGFR‐TKIs in *EGFR*‐mutated NSCLC cells.^13^ Therefore, the allele frequency of EGFR‐activating mutations might play a crucial role in predicting osimertinib sensitivity in tumors with acquired resistance harboring the *EGFR*‐T790M mutation. Further clinical investigations are needed to verify these findings.

The tumor heterogeneity was attributed to both genetic and non‐genetic mechanisms acting at various intervals for targeted drugs, including EGFR‐TKIs in NSCLC patients with EGFR mutations.^26^, ^27^ TMB, CIS, and MATH scores help to evaluate intratumor genetic heterogeneity, which is closely related to drug resistance and poor outcomes in patients with lung cancer harboring *EGFR* mutations.^28^, ^29^, ^30^, ^31^, ^32^, ^33^ In this study, gefitinib‐resistant cells showed diverse CIS, TMB, and MATH scores compared with dacomitinib‐induced resistant cells, suggesting that the wide range of inhibitory activity of dacomitinib might prevent gene alterations and yield a low proportion of sub‐clones with *EGFR*‐T790M mutation.^23^ The distribution of epigenetic intratumor heterogeneity contributes to insensitivity to EGFR‐TKIs in intrinsic and acquired resistance.^14^, ^32^, ^34^, ^35^, ^36^, ^37^, ^38^ T790M sub‐clonality has been associated with worse outcomes in osimertinib‐treated patients owing to the presence of other co‐occurring TKI‐resistant alterations.^39^ The regulation of EMT‐related proteins can be attributed to the ErbB2 and ErbB4 axis in *EGFR*‐T790M‐resistant cells.^40^, ^41^ Thus, management of the accumulation of “off‐target” resistant mechanisms, including those involving HER family proteins, plays a vital role in combating osimertinib sensitivity in tumors with acquired resistance harboring *EGFR*‐T790M mutation. The broad inhibitory activity of dacomitinib might reduce the accumulation of epigenetic changes. In addition, the long‐term exposure to dacomitinib enhanced the epithelial features compared with those of parental cells, suggesting that drug pressure by dacomitinib might accelerate sensitivity of the cells to osimertinib.

There are a few limitations to this study. First, the experiments were performed using a single parental cell line because we attempted to elucidate clonal evolution of T790M‐mutated tumors during treatment with different generations of EGFR‐TKIs. Second, the number of in vivo created resistant cells is limited—one strain from gefitinib‐resistant and two strains from dacomitinib‐resistant tumors. Therefore, we are not able to conclusively determine if our findings demonstrate a common and crucial behavior of resistant tumors with *EGFR*‐T790M mutation. Third, the biological difference in each resistant clone treated with osimertinib was limited. Further clinical and preclinical investigations are warranted to confirm our findings.

In summary, gefitinib‐resistant cells were less sensitive to osimertinib than dacomitinib‐resistant cells among NSCLC cells with EGFR‐T790M resistant mutation. It was caused by decreased dependency on EGFR signaling, related to increased tumor heterogeneity, such as increased TMB and EMT. Thus, treatment with different generations of EGFR‐TKIs might modulate heterogeneity among tumors with *EGFR*‐T790M mutation accompanied by genomic instability and activation of “off‐target” resistance mechanisms, resulting in reduced osimertinib sensitivity. The progression of tumor heterogeneity via genetic and non‐genetic mechanisms was associated with osimertinib sensitivity in tumors with acquired resistance harboring *EGFR*‐T790M mutation.

## CONFLICT OF INTEREST

T. Yamada received commercial research grants from Pfizer, Ono Pharmaceutical, Janssen Pharmaceutical K.K., and Takeda Pharmaceutical Company Limited. S. Yano obtained commercial research grants from Chugai Pharmaceutical and Boehringer‐Ingelheim, and has received speaking honoraria from Chugai Pharmaceutical, Boehringer‐Ingelheim, Novartis, and Pfizer. K. Takayama received research grants from Chugai‐Roche and Ono Pharmaceutical and personal fees from AstraZeneca, Chugai‐Roche, MSD‐Merck, Eli Lilly, Boehringer‐Ingelheim, and Daiichi‐Sankyo. No potential conflict of interest was disclosed by the other authors.

## AUTHOR CONTRIBUTIONS

Yuki Katayama: software, validation, investigation, visualization, and writing ‐ original draft; Tadaaki Yamada: conceptualization, resources, writing ‐ original draft, project administration, methodology, and funding acquisition; Shinsaku Tokuda: data curation; Naoko Okura: investigation; Naoya Nishioka: investigation; Kenji Morimoto: investigation; Keiko Tanimura: formal analysis; Yoshie Morimoto: validation; Masahiro Iwasaku: validation; Mano Horinaka: resources; Toshiyuki Sakai: resources; Kenji Kita: resources; Seiji Yano: resources; Koichi Takayama: supervision.

## ETHICS APPROVAL

This study was approved by the institutional review board at University Hospital, Kyoto Prefectural University of Medicine (approval no. M29‐529).

## Supporting information

Supplementary Material

## Data Availability

The data that support the findings of this study are available within the Supporting Information files and from the corresponding author upon reasonable request.
