# Supplementary material for: Heterogeneity among tumors with acquired resistance to EGFR tyrosine kinase inhibitors harboring EGFR‐T790M mutation in non‐small cell lung cancer cells
Source: Cancer Med. 2022 Jan 14;11(4):944–55. doi: 10.1002/cam4.4504 (PMC8855901; doi:10.1002/cam4.4504)
Supplement: Supplementary file 1 — Supplementary Material [file CAM4-11-944-s001.pdf]

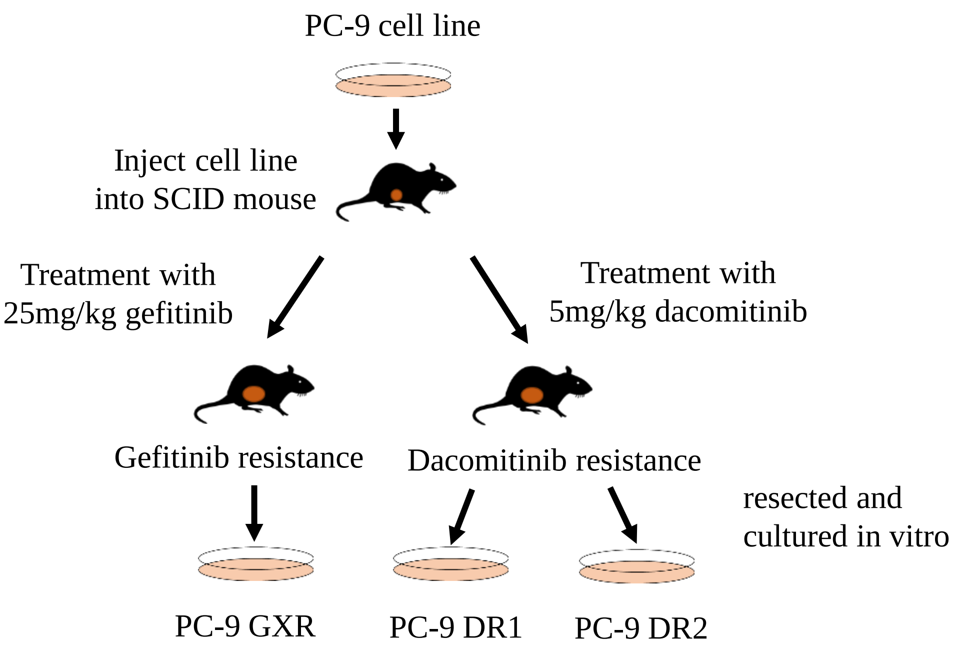

Supplementary Figure 1.
Establishment of gefitinib- or dacomitinib-resistant tumors using a mouse xenograft model.
A mouse xenograft model was developed from PC-9 cells with *EGFR* exon 19 deletion mutation. The subcutaneous tumors were continuously treated with gefitinib (25 mg/kg) or dacomitinib (5 mg/kg) until tumor regrowth. At the time of resistance acquisition, tumors were resected, and gefitinib-resistant cells (PC-9 GXR) and dacomitinib-resistant cells (PC-9 DR1 and DR2) were cultured in vitro.

A

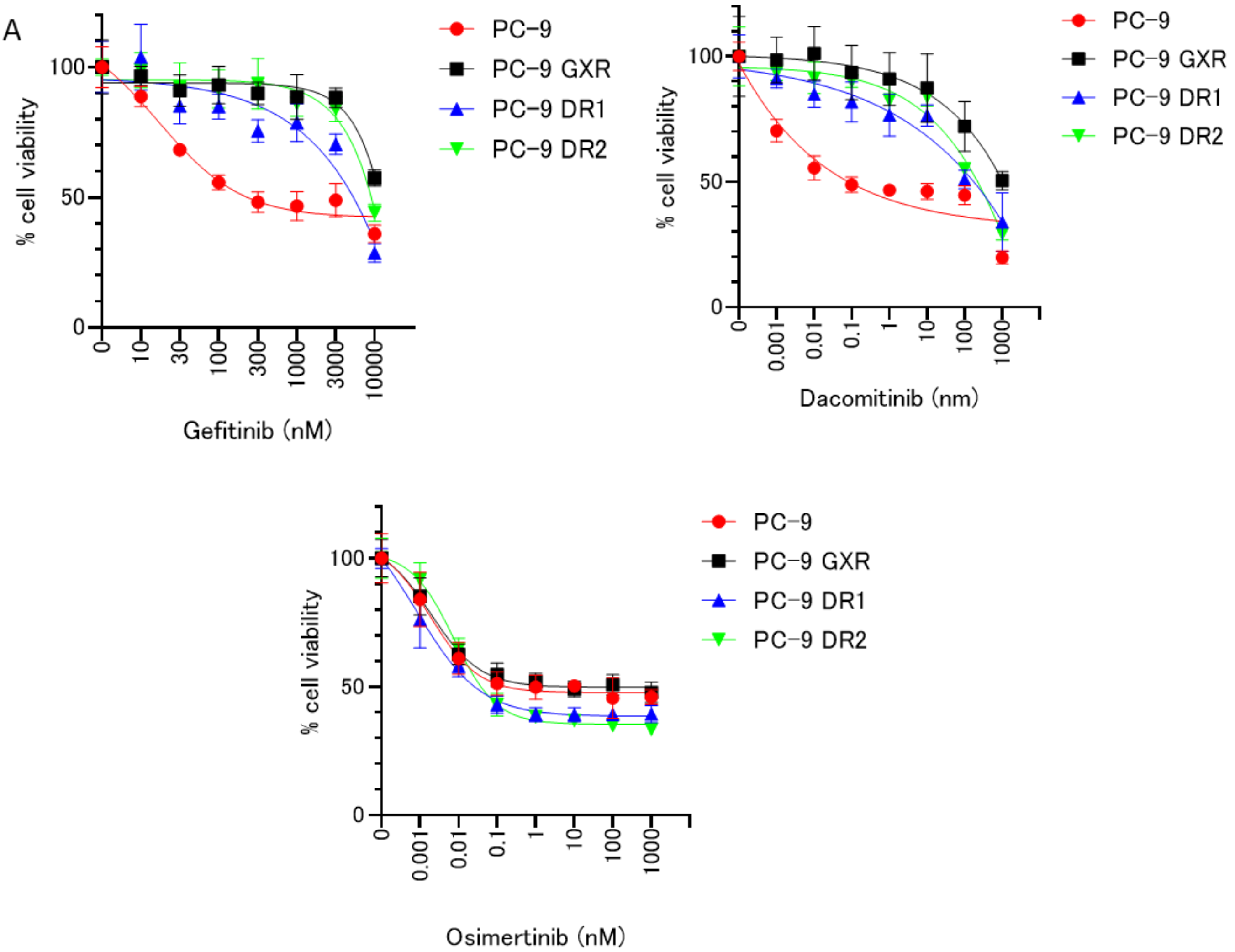

B

IC50 (nM)	PC-9	PC-9 GXR	PC-9 DR1	PC-9 DR2
Gefitinib	789.4	>10000	5068	9034
Dacomitinib	0.3794	1150	149.4	164.1
Osimertinib	102.6	173.5	17.73	23.05

Supplementary Figure 2.
Effect of EGFR-TKIs on the viability of PC-9 and resistant cells in vitro.
A, Viability of PC-9 and resistant cells after treatment with indicated concentrations of gefitinib, dacomitinib, and osimertinib for 72 h, determined by MTT assay. B, Concentrations of gefitinib, dacomitinib, and osimertinib inhibiting 50% of the growth (IC50) of PC-9 and resistant cells.

A

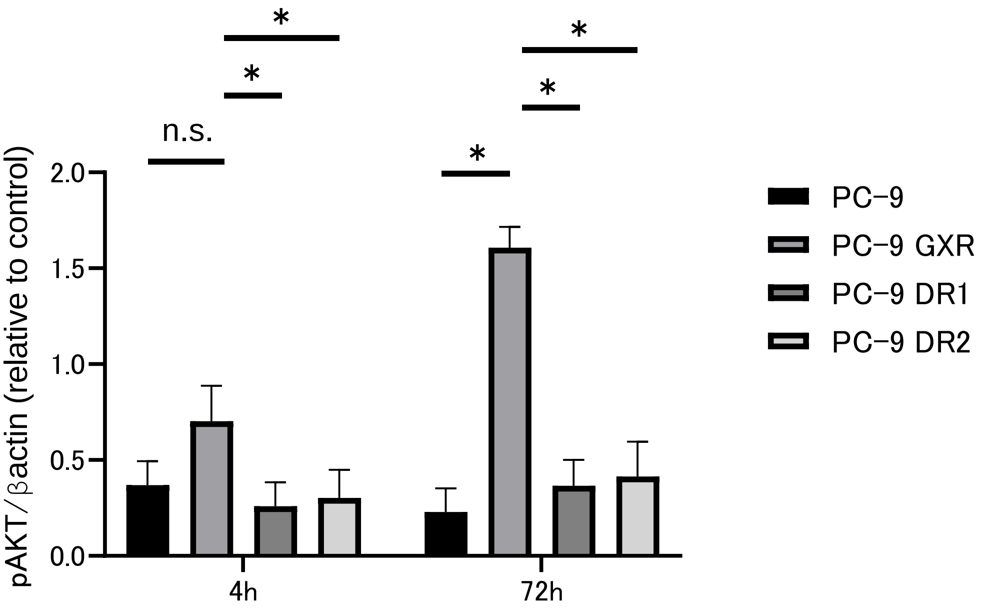

B

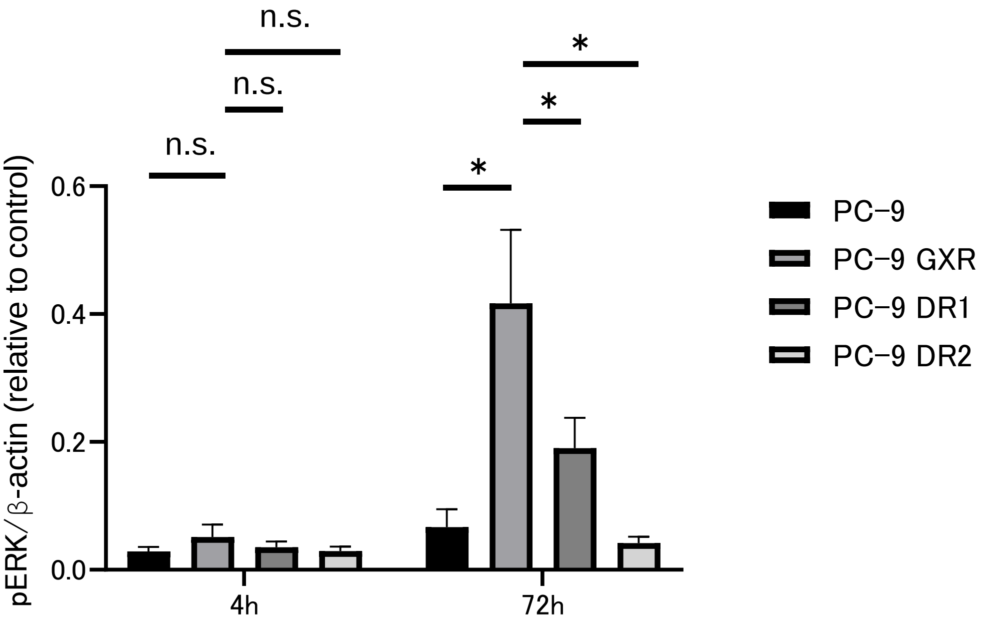

Supplementary Figure 3.
Quantification of AKT and ERK phosphorylation levels in PC-9 and resistant cells treated with osimertinib
Graphs showing the relative AKT (A) and ERK (B) phosphorylation levels following treatment with osimertinib (100 nmol/L) for 4 or 72 h in PC-9 and resistant cells. Relative AKT and ERK phosphorylation levels in Western blotting were quantified using ImageJ software. *$P < 0.05$ compared with PC-9 GXR cells treated with osimertinib, two-way ANOVA.

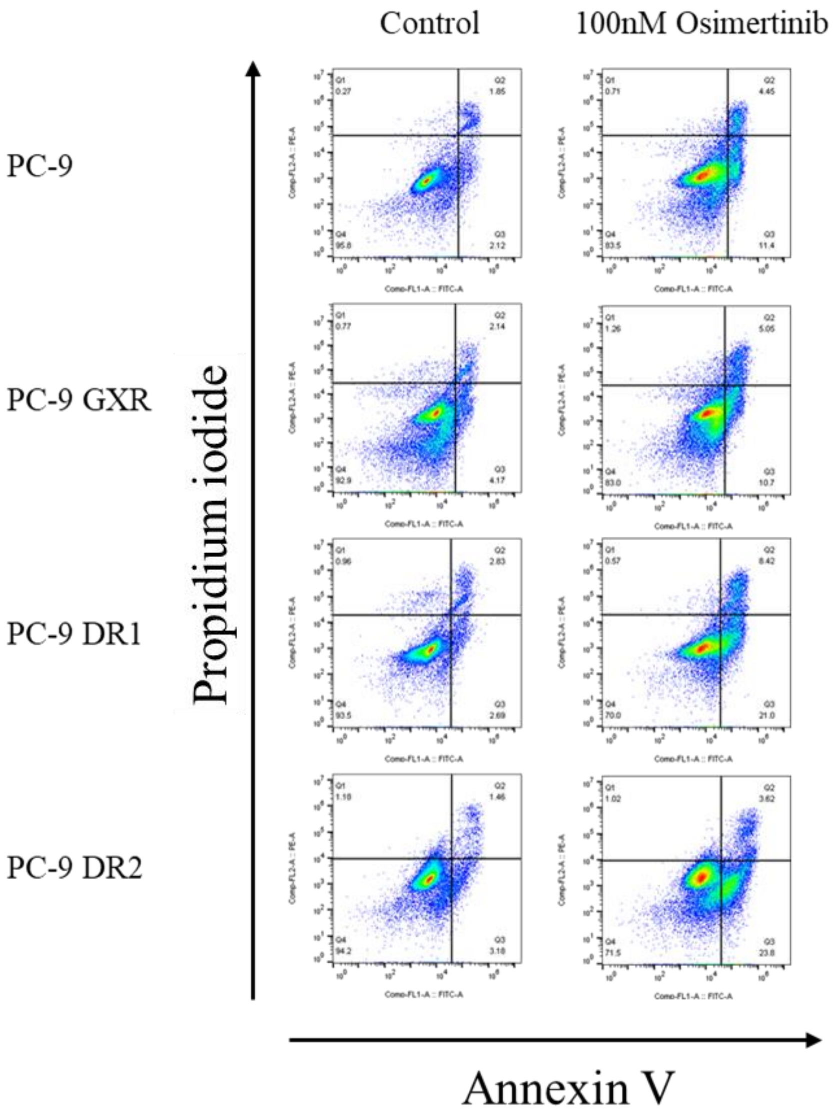

Supplementary Figure 4.
The proportions of apoptotic PC-9 and resistant cells after treatment with 100 nM osimertinib.
PC-9 and resistant cells were harvested 48 h after treatment with 100 nM osimertinib and washed twice with ice-cold PBS. Cells were collected and incubated with Annexin V-FITC and propidium iodide (PI) for 15 min at room temperature. The cells were analyzed using BD Accuri™ C6 Plus Flow Cytometer and FlowJo® software.

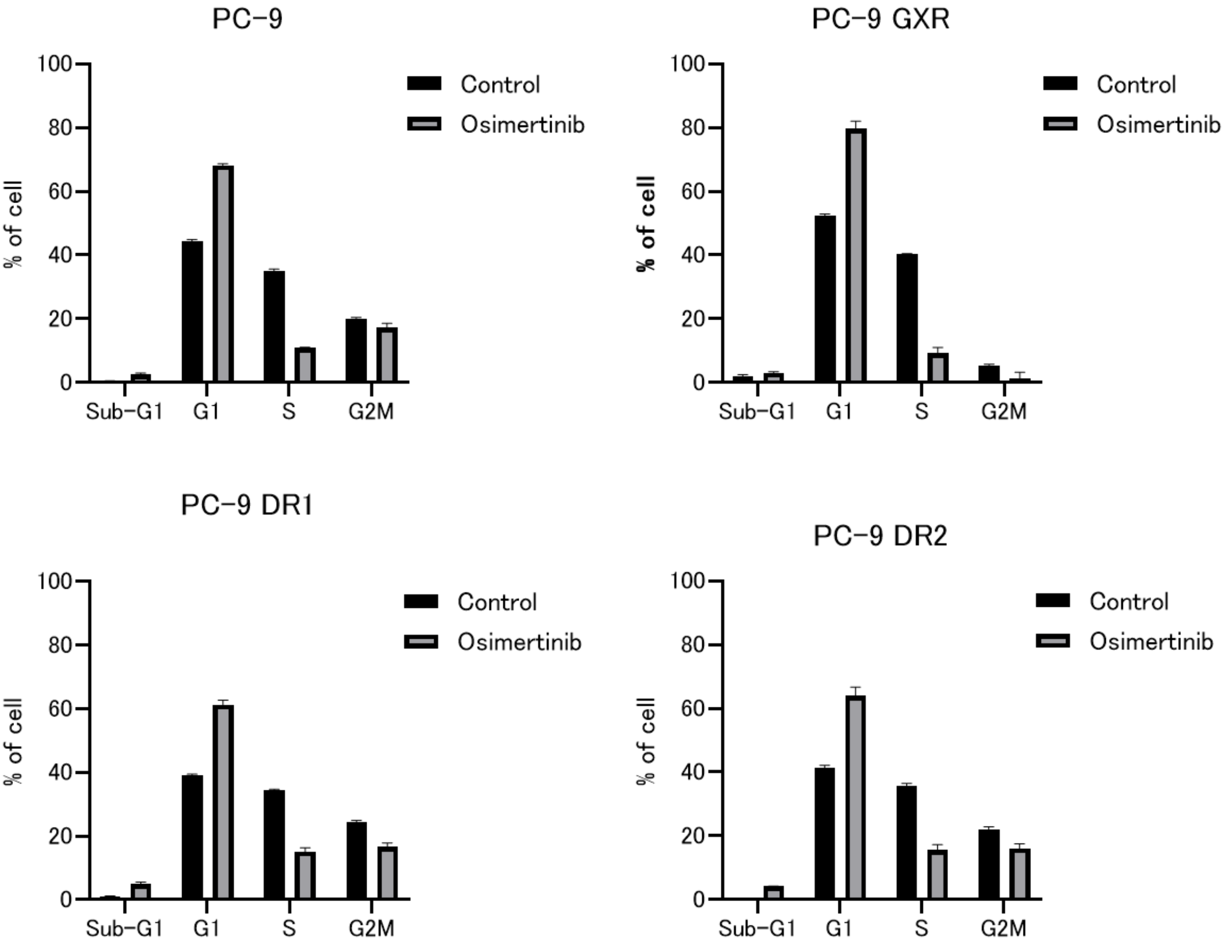

Supplementary Figure 5.

Distribution of PC-9 and resistant cells according to the phase of the cell cycle after treatment with 100 nM osimertinib.

PC-9 and resistant cells were harvested 48 h after treatment with 100 nM osimertinib and washed twice with ice-cold PBS. Cells were collected and resuspended in propidium iodide (PI) staining buffer, followed by analysis using BD Accuri™ C6 Plus Flow Cytometer and FlowJo® software.

A

A

Gene sets	size	Normalized enrichment score	p-value	False discovery rate
HALLMARK_INTERFERON_GAMMA_RESPONSE	193	1.95	< 0.001	0.005
HALLMARK_INTERFERON_ALPHA_RESPONSE	93	1.87	< 0.001	0.002
HALLMARK_EPITHELIAL_MESENCHYMAL_TRANSITION	195	1.84	< 0.001	0.003
HALLMARK_TGF_BETA_SIGNALING	53	1.82	0.003	0.004
HALLMARK_ALLOGRAFT_REJECTION	194	1.75	< 0.001	0.005
HALLMARK_G2M_CHECKPOINT	190	1.74	< 0.001	0.005
HALLMARK_TNFA_SIGNALING_VIA_NFKB	193	1.69	< 0.001	0.007

B

Gene sets	size	Normalized enrichment score	p-value	False discovery rate
HALLMARK_CHOLESTEROL_HOMEOSTASIS	70	2.55	< 0.001	< 0.001
HALLMARK_EPITHELIAL_MESENCHYMAL_TRANSITION	195	2.27	< 0.001	< 0.001
HALLMARK_MTORC1_SIGNALING	196	1.78	< 0.001	0.007
HALLMARK_MYOGENESIS	193	1.72	< 0.001	0.009
HALLMARK_ANDROGEN_RESPONSE	95	1.69	< 0.001	0.009
HALLMARK_UNFOLDED_PROTEIN_RESPONSE	103	1.62	0.004	0.018
HALLMARK_P53_PATHWAY	193	1.62	< 0.001	0.016
HALLMARK_TNFA_SIGNALING_VIA_NFKB	193	1.6	0.002	0.017
HALLMARK_UV_RESPONSE_DN	139	1.53	0.004	0.035

Supplementary Figure 6.
Gene set enrichment analysis (GSEA) of microarray data for PC-9 GXR versus PC-9 DR1 cells (A), and PC-9 GXR versus PC-9 DR2 cells (B).
The list of hallmark gene sets showed a ≥ 1.5-fold increase in normalized enrichment score for PC-9 GXR cells compared to those for PC-9 DR1 or DR2 cells.

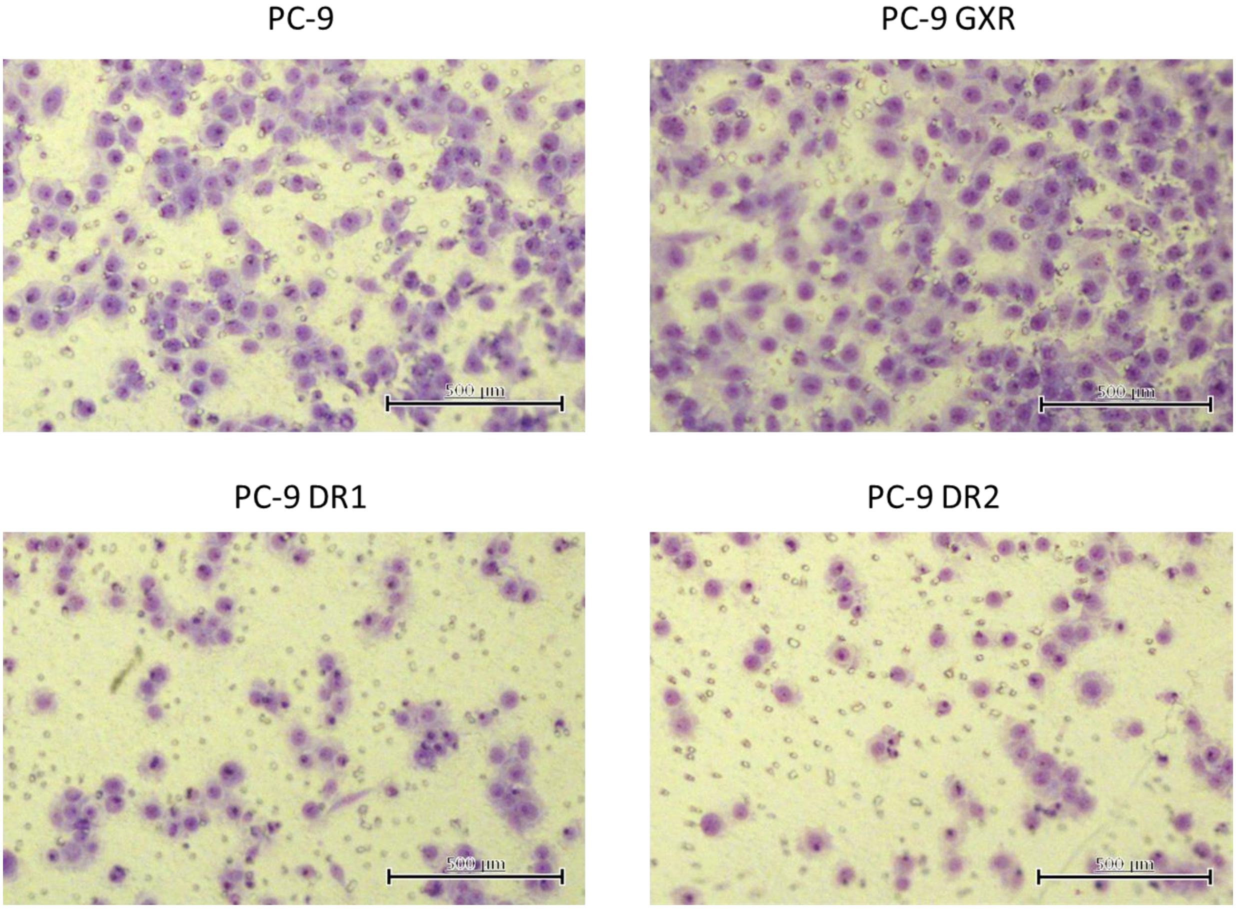

Supplementary Figure 7.
Migration assay for PC-9 and resistant cells
The migration ability of PC-9 and resistant cells was evaluated by migration assay. Cells were observed by bright field microscopy at ×100 magnification. Scale bar, 500 μm.

Table S1: Details of the antibodies used in this study

Antibodies	Dilution	Company	Catalog#
p-EGFR (Tyr1068)	1 : 1000	Cell Signaling Technology	3777
p-AKT (Ser473)	1 : 1000	Cell Signaling Technology	4060
t-AKT	1 : 1000	Cell Signaling Technology	9272
p-ERK1/2 (Thr202/Tyr204)	1 : 1000	Cell Signaling Technology	4370
t-ERK1/2	1 : 1000	Cell Signaling Technology	4695
β-actin	1 : 1000	Cell Signaling Technology	4970
E-cadherin	1 : 1000	Cell Signaling Technology	3195
N-cadherin	1 : 1000	Cell Signaling Technology	13116
vimentin	1 : 1000	Cell Signaling Technology	5741
t-EGFR	1 : 1000	R&D Systems	AF231
